# Sarcopenia Among Older Adults: Epidemiology, Proteomics, Technology and Biomarkers

**DOI:** 10.1093/geroni/igaf122.1216

**Published:** 2025-12-31

**Authors:** Josef Coresh, Shoshana Ballew, Jennifer Schrack

## Abstract

This session delves into research in sarcopenia, a condition characterized by the loss of muscle mass and function in older adults, from epidemiology, proteomics, technology and biomarkers. The session begins with a discussion on the challenges and methodologies in defining sarcopenia within a population-based study of the oldest adults, highlighting the importance of accurate and practical diagnostic criteria in understanding its prevalence and impact. Following this, data on the incidence of sarcopenia from age 66 to 90+ years, using the ARIC cohort from 2011 to 2023, is presented to provide insights into the age-related progression of sarcopenia and its public health implications. The session then explores the use of actigraphy monitoring among older adults with sarcopenia, showcasing innovative approaches to tracking physical activity patterns that offer new avenues for early detection and management. The proteomics of sarcopenia is examined next, focusing on both shared and distinct biological pathways that contribute to its prevalence and future risk, shedding light on the molecular underpinnings and potential targets for intervention. Finally, longitudinal changes in the creatinine muscle index, a novel blood marker of muscle mass, among older adults are discussed, emphasizing the importance of continuous monitoring and early intervention in mitigating the adverse effects of muscle loss. Overall, the session provides a wide-ranging set of results in sarcopenia research and a discussion of the implications of this common and treatable condition in aging populations.

